# A multilevel analysis of prevalence and factors associated with female child marriage in Nigeria using the 2018 Nigeria Demographic and Health Survey data

**DOI:** 10.1186/s12905-022-01733-x

**Published:** 2022-05-11

**Authors:** Obasanjo Afolabi Bolarinwa, Bright Opoku Ahinkorah, Joshua Okyere, Abdul-Aziz Seidu, Olalekan Seun Olagunju

**Affiliations:** 1 Department of Public Health Medicine, School of Nursing and Public Health, University of KwaZulu-Natal, Durban, South Africa; 2 Department of Demography and Social Statistics, Faculty of Social Sciences, Obafemi Awolowo University, Ile-Ife, Nigeria; 3 School of Public Health, Faculty of Health, University of Technology, Sydney, Australia; 4 Department of Population and Health, University of Cape Coast, Cape Coast, Ghana; 5 College of Public Health, Medical and Veterinary Services, James Cook University, Townsville, Australia

**Keywords:** Female child marriage, Prevalence, Factors, NDHS, Nigeria, Public health

## Abstract

**Background:**

Globally, there has been a decline in female child marriage (FCM) from 1 in 4 girls married a decade ago to approximately 1 in 5 currently. However, this decline is not homogenous because some regions are still experiencing a high prevalence of FCM. As such, the United Nations reiterated the need for concentrated efforts towards ending FCM to avoid more than 120 million girls getting married before their eighteenth birthday by 2030. Following this, we examined the prevalence and factors associated with FCM in Nigeria using multi-level analysis.

**Methods:**

We used cross-sectional data from the women’s file of the Nigeria Demographic and Health Survey (NDHS) conducted in 2018. A sample of 4143 young women aged 20–24 was included in the study. Our analysis involved descriptive, chi-square (χ^2^) and multi-level analyses. Results were presented in percentages, frequencies, and adjusted odds ratios (aOR) with their respective confidence intervals (CIs).

**Results:**

The prevalence of FCM in 2018 was 65.30%. Young Muslim women aged 20–24 [aOR = 1.40; 95% CI (4.73–7.52)], those with parity between one and two [aOR = 5.96, 95% CI 4.73–7.52], those residing in North East [aOR = 1.55; 95% CI (1.19–2.10)] and North West [aOR = 1.59; 95% CI (1.18–2.16)] had a higher odd of practicing FCM respondents with secondary education and above [aOR = 0.36; 95% CI (0.29–0.46)], those within the richer wealth index [aOR = 0.35; 95% CI (0.23–0.54)] and young women living in communities with high literacy level [aOR = 0.74; 95% CI (0.59–0.92)] were less likely to get married before age 18 years.

**Conclusion:**

Our findings indicate that FCM is high in Nigeria. Formal education, being rich and living in communities with high literacy levels were some protective factors that can be strengthened to ensure that FCM is reduced or eliminated in Nigeria. On the other hand, residing in North-East or North-West and having children between one and two were some prevailing factors that exacerbated the odds of experiencing FCM in Nigeria. Therefore, attention should be channelled towards mitigating these prevailing negative factors.

## Background

Providing equal opportunities for socio-economic development for both males and females is a topical issue now more than ever. Notwithstanding, there are some activities and practices that retards the realisation of equality. One of such developments is female child marriage (FCM). This phenomenon (FCM) refers to any formal marriage or informal union between a girl child below age 18 with an adult or another child with or without consent [1]. There has been a global decrease of FCM from 1 in 4 girls married a decade ago to approximately 1 in 5 now. However, this decline is not homogenous globally because some regions are still experiencing a high prevalence of FCM [2, 3].

FCM is a violation of the fundamental human rights of young women, and the importance of ending child marriage was highlighted in the Millennium Development Goals (MDGs), and now re-echoed in the Sustainable Development Goals (SDGs), particularly SDG 5.3, which envisions to eliminate child marriage by 2030. Therefore, to achieve this SDG target, there is a need for a yearly rate of reduction in the prevalence of FCM to 10% from about 23% [4]. This calls for pragmatic efforts and strategies that are backed by data and empirical evidence.

There are compelling evidences to show that FCM exposes girls to adverse health outcomes such as maternal morbidity, pregnancy and childbirth complications [5], and it is also the leading cause of high maternal mortality among adolescent girls [6]. Further adverse outcomes of FCM have also been linked to school dropout [7], poor sexual and reproductive health outcomes [8], low birth weight and a higher risk of preterm birth of about 30% [9, 10], poor child nutrition and health outcomes. Evidence from Ghana [11] reveals that FCM exacerbates girls' vulnerability to all forms of violence (physical, emotional, and sexual). In Nigeria, Braimah [12] reported that FCM results in a high prevalence of vesicovaginal fistula.

Given the harmful consequences associated with FCM, Nigeria has made some important moves to reduce the prevalence of FCM. In 2003, the country adopted the Child Rights Act (CRA), which clearly states the age of 18 as the minimum age for marriage in Nigeria [13]. Also, in 2015, Nigeria adopted another policy framework known as the Violence against Persons Prohibition Act (VAPPA). The role of the VAPPA is to address concerns about FCM in Nigeria, among other harmful practices like domestic violence and female genital cutting [13]. Notwithstanding all of these interventions and concerted efforts to eliminate FCM in Nigeria, there still remains a substantial proportion (about 40%) of girls who are married before the age of 18 [14].

Several factors (individual, household, and contextual) have been postulated to predict the occurrence of FCM [15]. Individual characteristics such as religion, ethnicity, parity, employment, exposure to mass media, and education attainment are noted to contribute substantially to FCM [16, 17]. Likewise, at the household level, poverty has been recognised as one of the key factors that affect FCM [12]. Parents who have low socioeconomic status are overwhelmed by the demands of their children. Hence, to get some relief from this burden, the female child is married off in reciprocity for financial support [12, 18].

Notable among the contextual issues that contribute to the perpetuation of FCM include the deep-rooted cultural norms, values, expectations and beliefs about FCM [12, 19]. Within the African culture, and most especially in Nigeria, FCM is seen as an appropriate cultural practice that can check premarital sex, promote chastity, and avert the shame of teenage pregnancy out of wedlock [12, 15]. Therefore, parents who marry off their female children before age 18 are accorded so much respect and prestige in the community, hence fuelling social acceptance of FCM [18]. Adebolewa [13] corroborates to this role of cultural norms in perpetuating FCM by emphasising that the strongly patriarchal nature of Nigeria’s norms reinforces FCM, as pre-arranged marriage can be done by a family to ensure political or socio-economic relations.

Bronfenbrenner [20] illustrated in social-ecological theory that continuous practice of female child marriage is the resilience interaction between child’s social and physical ecologies, which dictates the factors predicting children’s resilience to adversity [21]. However, children’s successful resilience to adversity often relies on individual, household or community factors beyond children’s control or capacity as opined by Cialdini, Kallgren [22] that these adversities are social norms that are rooted in one’s belief which could either be descriptive norms or injunctive norms [23]. Either of the norms has shown compliance to harmful practices such as female child marriage etc., despite individual negative attitudes towards the harmful practice [24]. For instance, a daughter or parent might have a negative attitude or not support marrying out a girl-child before age 18 in Nigeria, but the social norms of the household or community mandate such practice then the individual resilience of not complying with harmful practice became invalid.

Recently, FCM has been linked to female genital mutilation (FGM) in SSA and climatic vulnerability in Bangladesh[25, 26], while a study conducted in Mali argued that family size could influence FCM [27] however, this current study, therefore, examines the prevalence and factors associated with FCM practice in Nigeria using multi-level analysis of the latest Nigeria Demographic Health Survey (NDHS) conducted in 2018. This study is critical because, despite the high prevalence and overwhelming adverse health outcomes associated with FCM in Nigeria [14], no study has been able to account for the influence of individual and household/community-level factors contributing to the high prevalence using the latest NDHS dataset.

## Methods and materials

### Data source

The 2018 Nigeria Demographic Health Survey (DHS) was used for this study. DHS is a nationwide survey executed every five years. The surveys focused on key maternal and child health measures such as marital age, FGM, unintended pregnancy, skilled birth attendance, contraceptive use, intimate partner violence and immunization among under-fives. A stratified dual-stage sampling approach was employed during the survey to collect information on the respondents. Furthermore, a cluster sampling process (i.e., enumeration areas [EAs]) was involved in the survey, followed by systematic household sampling within the chosen EAs. The sample framework generally excludes nomadic and institutional groups, such as inmates and hotel occupants [28]. The women’s files with responses by women aged 15–49 were accessed [29]; however, this study participant was limited to young women between the age of 20–24 as inclusion criteria [3, 30].

### Sample size and inclusion criteria

The eligible sample size for this study was 4,543 women aged 20 to 24. The study included only respondents who had complete information on the variables of interest.

### Study variables

#### Outcome variable

The outcome variable for this study was “Age at first marriage”. To derive this variable, respondents were asked the age at which they first got married. Respondents were categorized as married before age 18 years if the response falls between age 1 to 17 and categorized as married 18 years and above if respondent’s response falls between 18 years and above [1, 31, 32].

#### Explanatory variables

Eleven explanatory variables were considered in this study and were grouped into individual-level variables and household/community level variables. These variables were determined by priori from previously published studies and the availability of the variables of interest in the datasets before the selection of all the explanatory variables [33, 34].

#### Individual-level factors

The individual-level factors were educational level, working status, religious, ethnicity, ever circumcised, parity (children ever born), and media exposure. Educational level was coded as ‘no education,’ ‘primary education’, and ‘secondary/higher education,’ while ‘working’ and ‘not working’ were the categories for working status. Religious were recoded as “Islam”, “Christianity” and “Traditionalist/others”, ethnicity was recoded into three major ethnic groups in Nigeria, namely, “Yoruba”, “Igbo”, “Hausa” while the remaining tribes were coded as others. Ever circumcised was coded as “Yes” for young women who were circumcised and “No” for those that were not circumcised, while parity was coded as “No child” for young women with any child, “1–3” for young women with children between 1 and 3, “4–6” for young women between 4–6. ‘Frequency of reading newspaper/magazine, listening to the radio and watching television were recategorized as media exposure. Those who didn’t engage in any of these were coded ‘No’ while those exposed to at least one of these were coded ‘Yes’.

#### Household/community-level factors

The household/community level variables were the place of residence, region, wealth quintile, and sex of the household head. These selected household/community level variables were based on their categorization in the DHS [35]. Place of residence was coded as ‘urban’ and rural, the Sex of the household head was coded as ‘male’ and ‘female’ while the wealth quintile was computed using the standard DHS data on household ownership by selecting properties such as bicycles, house building materials, television, type of access to water and sanitation facilities. To make households on a continuous relative wealth scale, a composite variable, wealth status, was generated from these assets through Principal Component Analysis (PCA), and we divided the households into five quintiles of wealth: poorest, poorer, middle, wealthier, and wealthiest [36].

### Data analyses

The analyses began with a descriptive analysis table to display the prevalence of age at first marriage, and a chi-square test of independence (χ^2^) was used to show the association between age at marriage and the explanatory variables (see Table 1).

**Table 1:** Distribution of age at first marriage by explanatory variables

Variables	Frequency (n)	Percentage (%)	Age at first marriage (%)		p-value (χ^2^)
Individual-level variables			18 years and above	Below 18 years	
Educational level					p < 0.001
No education	2,104	46.33	15.23	84.77	
Primary	620	13.65	27.05	72.95	
Secondary and above	1,818	40.03	59.84	40.16	
Currently working					p < 0.001
Unemployed	2,024	44.55	30.11	69.89	
Employed	2,519	55.45	38.38	61.62	
Religious affiliation					p < 0.001
Christianity	1,299	28.59	61.05	38.95	
Islam	3,222	70.93	24.17	75.83	
Traditionalist and others	21	0.48	20.81	79.19	
Ethnicity					p < 0.001
Hausa	2,428	53.44	18.13	81.87	
Yoruba	400	8.82	74.07	25.93	
Igbo	311	6.86	74.29	25.71	
Others	1,403	30.89	43.33	56.67	
Ever circumcised					P < 0.05
No	4,370	96.18	34.19	65.81	
Yes	173	3.82	47.54	52.46	
Parity					p < 0.001
None	595	13.10	63.11	36.89	
1–3	3,743	82.39	31.89	68.11	
4–6	204	4.51	3.51	96.49	
Media exposure					p < 0.001
No	1,921	42.29	20.33	79.67	
Yes	2,622	57.71	45.22	54.78	
Household/community level variables					
Place of residence					p < 0.001
Urban	1,427	31.43	52.21	47.79	
Rural	3,115	68.57	26.67	73.33	
Wealth index					p < 0.001
Poorest	1,094	24.09	16.27	83.73	
Poorer	1,186	26.12	21.15	78.05	
Middle	990	21.79	35.95	64.05	
Richer	808	17.70	56.54	43.46	
Richest	463	10.21	72.10	27.90	p < 0.001
Sex of household head					
Male	4,174	91.88	33.39	66.61	
Female	369	8.12	49.44	50.56	
Region					p < 0.001
North Central	696	15.33	46.86	53.14	
North East	927	20.42	21.91	78.09	
North West	1907	41.99	18.64	81.36	
South East	264	5.82	73.76	26.24	
South South	293	6.46	59.59	40.41	
South West	453	9.98	70.82	29.18	
Community literacy level					p < 0.001
Low	2,327	51.23	21.81	78.19	
Medium	739	16.28	29.33	70.67	
High	1,476	32.49	57.70	42.30	
Community socioeconomic status					p < 0.001
Low	3,527	77.63	28.11	71.89	
High	1,016	22.37	57.55	42.45	
Nigeria	n = 4543	100	34.70	65.30	

Weighted NDHS, 2018

### Modelling approaches

A multilevel logistic regression model (MLRM) was used to examine the association between the individual and household/community factors and age at marriage in Nigeria using the recent DHS dataset. The Stata command “melogit” was used in fitting these models. A 2-level model for binary responses was specified, reporting age at marriage below 18 or not for young women aged 20–24. At the first level, women were modelled from households (Individual level) and at the second level, households were modelled from PSUs (household/community levels). Four models were constructed in this study. The first model was the empty model/null model (Model 0), which is the model that shows the variance in the outcome variable, which is the age at marriage, attributed to the clustering of primary sampling units (PSUs), however, this model has no explanatory variable included. The second model contained only the individual-level factors (Model I), while the third model contained the household/community-level factors (Model II). The final model was the complete model (Model III) that simultaneously controlled for the individual and household/community factors.

The MLRM consists of fixed and random effects [37, 38]. The fixed effects (measures of association) showed results of the association between the selected explanatory variables and the outcome variable (age at marriage) and were reported as adjusted odds ratios (aOR) with their 95% confidence intervals (CIs), while the random effects (measures of variations) were assessed using Intra-Cluster Correlation (ICC) [39]. This means that variations in factors influencing FCM in Nigeria were drawn from within PSUs, which enabled us to draw an appropriate conclusion [40]. The LR test was used to check for model adequacy. Both Akaike’s Information Criterion (AIC) and Bayesian Information Criteria (BIC) were used to measure how well the different models fitted the data. The sample weight (v005/1,000,000) was applied to correct for over-and under-sampling, while the ‘svy’ command was used to account for the complex survey design and generalizability of the findings. The analyses were carried out with Stata version 16.0 (Stata Corporation, College Station, TX, USA).

### Ethical approval

Ethical approval was granted by the Institutional Review Board of ICF International, and the DHS Program approved the use of the dataset for this study, which the dataset is freely available at https://dhsprogram.com/data/available-datasets.cfm upon request. Individual informed consent was sought from all participants during data collection. All methods were performed according to the relevant guidelines and regulations in line with the World Medical Association Declaration of Helsinki Ethical principles [41].

## Results

### Descriptive results

The prevalence of FCM in 2018 was 65.30%. At the individual level, women with no education (84.77%), unemployed (69.89%), traditionalist and other religious (79.19%), Hausa ethnicity (81.87) and those with 4–6 children (96.49%), and those unexposed to mass media (79.67%) had a higher prevalence of getting married before 18 years. There were also variations in the prevalence of girl child marriage across the various household/community factors. Young women residing in the rural area (73.33%), those within the poorest wealth quintile (83.73%), those in male-headed households (66.61%), those residing in the North-West region (81.36%), young women residing in the community with low literacy level (78.19%), and those residing in the community with low socioeconomic status (71.89%) had a higher prevalence of girl child marriage. All the individual and household/community factors showed statistically significant associations with age at marriage.

### Fixed effects (measures of associations) results

With the individual-level factors, the likelihood of getting married below the age of 18 years was lower among young women who had secondary education & above [aOR = 0.36; 95%(CI 0.29–0.46)], and those from the Yoruba ethnic group [aOR = 0.37; 95%(CI 0.22–0.62)], compared to young women who were not educated, and those from Hausa ethnic group. While those with parity of 1–3 children [aOR = 5.96; 95%(CI 4.73–7.52)] and young women practicing Islam [aOR = 1.40; 95%(CI 1.09–1.81)] were more likely to get married below age 18 years compared to young women with no child and those practicing Christianity.

In terms of the household/community factors, young women within the richer wealth index [aOR = 0.35; 95%(CI 0.23–0.54)], and those living in communities with high literacy level [aOR = 0.74; 95%(CI 0.59–0.92)] were less likely to get married below 18 years compared to young women within poorest wealth index, and those residing in a community with low literacy level. While those residing in North East region [aOR = 1.55; 95%(CI 1.19–2.10)] and North West region [aOR = 1.59; 95%(CI 1.18–2.16)] were more likely to get married below age 18 years compared to young women residing in North-Central region.

### Random effects (measures of variations) results

As shown below in Table 2, the empty model depicted a substantial variation in the likelihood of female child marriage in Nigeria across the PSUs clustering [σ2 = 1.88; 95%(CI 1.50–2.34)]. The empty model (Model 0) indicated that 36% of the variation in age child marriage in Nigeria was attributed to the variation between-cluster characteristics, i.e., (ICC = 0.36). The variation between-cluster decreased to 6% in Model I, representing only the individual level model (Model I). In the community-level only model (Model II), the ICC increased to 8% from the previous ICC of 6% at the individual level model. While the complete model (Model III), with both the individual and household/community models, ICC declined to 5%. This further reiterates that the variations in the likelihood of FCM in Nigeria are attributed to the clustering differences within PSUs. The AIC and BIC values showed a successive reduction, which means a substantial improvement in each of the models over the previous model and also affirmed the goodness of Model III developed in the analysis. Therefore, the complete model (Model III) with both the selected individual and household/community factors was chosen to predict female child marriage's eventuality or occurrence.

**Table 2:** Multilevel logistic regression models for individual and household/community factors associated with girl child marriage

Variables	Model 0	Model I	Model II	Model III
		aOR[95% CI]	aOR[95% CI]	aOR[95% CI]
Fixed effects results				
Individual-level variables				
Educational level				
No education		RC		RC
Primary		0.63***[0.49–0.81]		0.75*[0.58–0.97]
Secondary and above		0.24***[0.20–0.30]		0.36***[0.29–0.46]
Currently working				
Unemployed		RC		RC
Employed		1.23*[1.04–1.45]		1.16 [0.98–1.37]
Religious affiliation				
Christianity		RC		RC
Islam		1.44**[1.15–1.80]		1.40**[1.09–1.81]
Traditionalist and others		1.65 [0.70–3.88]		1.52 [0.63–3.70]
Ethnicity				
Hausa		RC		RC
Yoruba		0.17***[0.13–0.25]		0.37***[0.22–0.62]
Igbo		0.20***[0.14–0.29]		0.41**[0.23–0.74]
Others		0.46***[0.37–0.57]		0.53***[0.41–0.68]
Ever circumcised				
No		RC		RC
Yes		0.75 [0.49–1.16]		0.80 [0.52–1.24]
Parity				
None		RC		RC
1–3		5.94***[4.74–7.46]		5.96***[4.73–7.52]
4–6		50.54***[25.13–101.66]		52.39***[25.91–105.92]
Media exposure				
No		RC		RC
Yes		0.78**[0.84–1.71]		0.96 [0.80–1.16]
Household/community level variables				
Place of residence				
Urban			RC	RC
Rural			0.90 [0.73–1.11]	1.00 [0.80–1.24]
Wealth index				
Poorest			RC	RC
Poorer			0.92 [0.73–1.16]	1.14 [0.90–1.46]
Middle			0.62***[0.49–0.80]	0.86 [0.66–1.12]
Richer			0.38***[0.29–0.50]	0.59**[0.43–0.80]
Richest			0.18***[0.12–0.27]	0.35***[0.23–0.54]
Sex of household head				
Male			RC	RC
Female			0.71*[0.54–0.92]	0.84 [0.64–1.11]
Region				
North Central			RC	RC
North East			2.40***[1.89–3.06]	1.55**[1.19–2.10]
North West			3.35***[2.66–4.21]	1.59**[1.18–2.16]
South East			0.48***[0.34–0.68]	0.73 [0.40–1.33]
South South			1.01 [0.74–1.38]	1.31 [0.94–1.82]
South West			0.60**[0.44–0.82]	0.80 [0.50–1.27]
Community literacy level				
Low			RC	RC
Medium			0.67**[0.52–0.86]	0.74*[0.57–0.95]
High			0.48***[0.39–0.59]	0.74**[0.59–0.92]
Community socioeconomic status				
Low			RC	RC
High			1.21 [0.92–1.58]	1.04 [0.79–1.37]
Random effects results				
PSU variance (95% CI)	1.88 [1.50–2.34]	0.20 [0.09–0.45]	0.28 [0.15–0.49]	0.16 [0.06–0.43]
ICC	0.36	0.06	0.08	0.05
LR test	χ^2^ = 346.66, p < 0.001	χ^2^ = 8.06, p < 0.05	χ^2^ = 17.60, p < 0.001	χ^2^ = 5.06, p < 0.05
Wald χ^2^	Reference	852.30***	649.36***	838.61***
Model fitness				
Log-likelihood	− 2759.72	− 2192.48	− 2396.81	− 2141.45
AIC	5523.45	4412.95	4825.62	4338.91
BIC	5536.28	4502.77	4928.26	4518.53
Number of clusters	1191	1191	1191	1191

Weighted NDHS, 2018

Exponentiated coefficients; 95% confidence intervals in brackets; AOR: adjusted Odds Ratios; CI: Confidence Interval; RC: Reference Category

^*^p < 0.05; **p < 0.01; ***p < 0.001

AIC: Akaike’s Information Criterion; BIC: Schwarz’s Bayesian Information Criteria; PSU: Primary Sampling Unit; ICC: Intra-Class Correlation; LR Test: Likelihood ratio Test

Model 0 is the null model, a baseline model without any determinant variable

The model I is adjusted for individual-level variables (Educational level, currently working, Religious, Ethnicity ever circumcised, parity and media exposure)

Model II is adjusted for household/community level variables (Place of residence, region, wealth index and sex of household head)

Model III is the final model adjusted for both individual and household/community level variables

## Discussion

FCM is an issue that has attracted the attention of the global community due to its importance in maternal health. Nigeria has been identified among the countries with a high prevalence of FCM [42]. Moreover, studies have shown that if left unabated, FCM could potentially retard the attainment of the SDGs, specifically, SDG 1 (end poverty in all its forms everywhere), 2 (ensure healthy lives and promote well-being for all at all ages), 3 (ensure inclusive and equitable quality education) and 4 (gender equality and empower all women and girls). Against this background and the imperativeness to understand the determinants of FCM in Nigeria, we investigated this phenomenon. Our findings show a high prevalence of FCM in Nigeria in 2018, with girls with parity of 1–3 children had a higher likelihood of being married before the age of 18. This is worrying because despite all concerted efforts through legislations, and policies (e.g., CRA, VAPPA) to eliminate FCM in Nigeria [13], more than half of the proportion of the women who participated in the study had been married before age 18.

Results from our study revealed that the place of residence was significantly associated with the odds of experiencing FCM. It is indicative from the study that females in rural areas have a greater likelihood of experiencing FCM. A similar finding was reported by Workineh, Kibretb and Degu [43], who found that the odds of FCM were more pervasive among rural residents than the urbanite counterparts. In the same vein, this observation is consistent with the findings from Pankhurst et al. [44] that showed that rural residents had a higher likelihood of experiencing FCM compared to their urban-dwelling counterparts. A possible explanation for this finding may be that urban residents, unlike rural residents, may be exposed to female empowerment programmes and interventions and higher education, thereby shaping their thoughts and motives towards the girl child's education rather than sending these girls into marriage.

Our study revealed that having secondary or higher education was associated with lower odds of experiencing FCM compared to those with no education. The finding is consistent with several findings from Nigeria and across the globe [3, 45–50]. For instance, Mpilambo et al. [48] found a negative, significant association between formal education and early marriage among young women in Congo. The current findings iterate that education is a protective factor for FCM. This implies that one of the most effective ways to combat FCM in Nigeria is to rejuvenate and strengthen girls' formal education. Formal education at any level has significant potential in reducing the likelihood of FCM. Moreover, Envuladu et al. [45] postulate that educated parents are more likely to delay the marriage of their young female children than those with no education. Therefore, if girl child education in Nigeria is given the maximum priority, the country will be moving towards attaining the SDGs and significantly winning the fight against FCM.

Concerning the effect of religion on the likelihood of FCM, we found that young women who practiced Islam had a higher likelihood of FCM compared to those who practiced Christianity. This is worth noting as Nigeria is a country where religion contributes significantly to the formation of values, norms and practices at both the micro (individual and family) and macro-level [46]. Our finding, thus, accords with an earlier study by Braimah [12], which posits that in Nigeria, particularly in Northern Nigeria, where Islam is predominant, there is a high prevalence of female child marriages. The author continues by stating that the reason for the high likelihood of FCM among Muslims in Nigeria is grounded on the fact that, in the Hadiths, it is reported that the Prophet Muhammad married Aisha at the age of 9; therefore, justifying FCM in such communities [12].

Our study further revealed that the girls from the Yoruba ethnic group were less likely to marry before age 18 compared to the girls who belonged to the Hausa ethnic group. This corroborates an earlier study by Adebowale [13] that has also found people from the Yoruba ethnic group to be less likely to marry before age 18. A plausible explanation for this outcome could be attributed to the location of the Yoruba ethnic group. Predominantly, the Yoruba are found in the South-West of Nigeria, where most of them profess Christianity and have higher education levels compared to their counterparts from the Hausa ethnic group who are predominantly Muslim low or no formal education [13, 51].

At the community level, our findings indicate that compared to young women within the poorer wealth index, those in the richer wealth index had a lower likelihood of FCM. The result aligns with previous studies, such as Pankhurst et al. [44], who reported that poverty was a significant determinant of FCM as it increased the probability of experiencing child marriage. A similar finding was reported by Rumble et al. [3], where wealth was inversely associated with child marriage odds. Again, the result is substantiated by Corno and Voena [52], who posit that individuals from poor households find it difficult to secure credit facilities/market and, therefore, depend heavily on bride price for daily living. This may prompt parents of girls within the poorer wealth index to marry off their girl child in an attempt to reduce the financial burden associated with the upbringing of the child. Thus, corroborating the postulate that poverty is a critical driver of FCM.

Our study indicates that young women residing in the North East and North West of Nigeria had a significantly higher likelihood of marrying before age 18 compared to young women in the North Central region of Nigeria. This finding is consistent with Mobolaji, Fatusi and Adedini [46] that found Northern Nigeria to be associated with a higher likelihood of FCM. Likewise, the finding is supported by Grijns and Horii [53], who found that holding on to conservative Islamic perspectives increases a girl’s odds of marrying before age 18. This is grounded on the premise that the Hausa-Fulanis reside in Northern Nigeria and are predominantly Muslims and are influenced by Muhammad’s marriage to Aisha when she was just a child [12]. This finding also emphasizes the entrenching gender inequality and deep-rooted patriarchal systems that facilitate and spearhead FCM in these regions [54]. Beyond these justifications, Braimah [12] indicates that FCM is perceived as a conduit for preserving community virtues concerning the female child in Northern Nigeria. Thus, FCM is practiced in this region to promote chastity, cherish virginity, and prevent birth out of wedlock.

We found that young women residing in communities with high literacy levels were less likely to marry before age 18. Female child marriage is not just a social issue but an issue; it is also a serious cultural issue. As such, it is reinforced by long-standing socio-cultural norms, values, and beliefs. Through the enhancement of community literacy, certain misconceptions and myths concerning FCM. Hence, exacerbating the likelihood of young women being married before age 18. This is congruent to earlier findings that improving community literacy empowers individuals in the community and significantly facilitates the changing of retrogressive and regressive norms that fuel FCM [53].

Overall, findings from our study showed the importance of household/community level factors in FCM. This was evident from the 28% variations PSUs attributed to the household/community level predictors of FCM, which was higher than the 20% variations in PSUs attributed to the individual level predictors of FCM and the 16% attributed to all the factors. The household/community level factors also explained the greatest percentage of ICC of 8% compared to the individual level factors and all the factors together. This indicates that household/community factors are the major factors associated with FCM. Similar findings have been obtained in previous studies on the same subject [55–57].

### Strengths and limitations

While this study used a nationally representative dataset from NDHS, which grants it the statistical power to make a generalisation of Nigeria, the study was limited in some capacities. The use of secondary data restricts the analytical possibilities and thereby limits the analysis to the association. In effect, causal inferences cannot be made from this study. Moreover, the data used was self-reported data, which is prone to some biases; however, it was outside the team's powers to validate the dataset's responses. Also, the use of secondary data, which was quantitative, does not provide a holistic comprehension of the predictors of female child marriages in Nigeria. We were unable to analyse the effect of cultural norms and beliefs on FCM since we used a secondary dataset that did not cover such issues. Another limitation of our study is that apart from ever circumcised, the rest of the independent variables measured the current status of young women while the outcome variable (age at first marriage) may have occurred in the past for some of the respondents. Hence, there is the likelihood of reverse causality between the associated factors and the outcome variables. Therefore, interpretation of the findings needs to be done, taking into consideration this key limitation.

### Policy implication

The findings of this study have serious implications for policy and practice. Regarding policy, the findings that show the significant association between education and FCM bring to the fore the expedient need for the Federal Republic of Nigeria to review its educational policies to emphasize more on free and compulsory girl-child education at all levels. By doing so, the girls will be empowered to say no to FCM. More interventions should be titled to decrease the financial burden associated with educating the girl child. Moreover, we believe that the government, non-governmental organisations (NGOs) and community support officers (CSOs) must work collaboratively to significantly reduce unemployment and poverty in Nigeria. This will help parents have enough money to cater to their family rather than rely on their girl-child to marry to bring home some money. In the same vein, policies that will categorically discourage girl-child marriage about Nigerians practicing Islam should be considered with much support and emancipation by the religious leaders.

### Recommendations and research implication

We recommend that future studies explore a mixed-method approach where qualitative data will be collected and analysed to provide in-depth interpretations and understanding of the determinants of FCM in Nigeria, particularly why women in Northern Nigeria are more likely to marry before age 18. Also, future studies should consider ethnographic, contextual, and ecosystem-based research.

## Conclusion

Our findings suggest that in order for policymakers in Nigeria to effectively reduce or eliminate female child marriage, it is imperative to know its associated factors. This study examined the prevailing determinants contributing to the high prevalence of female child marriage practice in Nigeria using the latest DHS conducted in 2018. Our findings indicated that having secondary or higher education and community literacy were some protective factors that can be strengthened to ensure that FCM is reduced or eliminated in Nigeria. On the other hand, low socio-economic status, residing in Northern Nigeria, and low educational level are some modifiable factors that exacerbate the odds of experiencing FCM in Nigeria. Therefore, priority attention should be channelled towards the mitigation of the negative modifiable factors to augment the contribution of the protective factors towards the realisation of SDGs 1, 2, 3 & 4.

## Data Availability

The dataset can be accessed at https://dhsprogram.com/data/available-datasets.cfm.

## Abbreviations

FCM: Female Child Marriage
FGM: Female Genital Mutilation
DHS: Demographic Health Survey
NDHS: Nigeria Demographic Health Survey
AOR: Adjusted Odds Ratio
COR: Crude Odds Ratio
CI: Confidence Intervals
SDG: Sustainable Development Goals
UN: United Nations
LIMCS: Low-and-Middle-Income Countries
EAs: Enumeration Areas
SVY: Survey Command
TV: Television
NGOs: Non-governmental organisations
CSOs: Community Support Officers
